# Techniques of Human Embryonic Stem Cell and Induced Pluripotent Stem Cell Derivation

**DOI:** 10.1007/s00005-016-0385-y

**Published:** 2016-03-03

**Authors:** Jarosław Lewandowski, Maciej Kurpisz

**Affiliations:** Department of Reproductive Biology and Stem Cells, Institute of Human Genetics, Polish Academy of Sciences, Strzeszynska 32, 60-479 Poznan, Poland

**Keywords:** Stem cells, Cardiomyocytes, Pluri potency, Directed differentiation

## Abstract

Developing procedures for the derivation of human pluripotent stem cells (PSCs) gave rise to novel pathways into regenerative medicine research. For many years, stem cells have attracted attention as a potentially unlimited cell source for cellular therapy in neurodegenerative disorders, cardiovascular diseases, and spinal cord injuries, for example. In these studies, adult stem cells were insufficient; therefore, many attempts were made to obtain PSCs by other means. This review discusses key issues concerning the techniques of pluripotent cell acquisition. Technical and ethical issues hindered the medical use of somatic cell nuclear transfer and embryonic stem cells. Therefore, induced PSCs (iPSCs) emerged as a powerful technique with great potential for clinical applications, patient-specific disease modelling and pharmaceutical studies. The replacement of viral vectors or the administration of analogous proteins or chemical compounds during cell reprogramming are modifications designed to reduce tumorigenesis risk and to augment the procedure efficiency. Intensified analysis of new PSC lines revealed other barriers to overcome, such as epigenetic memory, disparity between human and mouse pluripotency, and variable response to differentiation of some iPSC lines. Thus, multidimensional verification must be conducted to fulfil strict clinical-grade requirements. Nevertheless, the first clinical trials in patients with spinal cord injury and macular dystrophy were recently carried out with differentiated iPSCs, encouraging alternative strategies for potential autologous cellular therapies.

## Introduction

The primary potential of stem cells lies is their self-renewal abilities and their possibility of giving rise to approximately 220 different types of specialized cells. In stem cell hierarchy, totipotent cells have an unlimited differentiation spectrum—they are able to form both the embryo and the placenta. In turn, pluripotent cells differentiate into all three embryonic germ layers: the endoderm, leading to the development of gastrointestinal tract, stomach and lungs; the mesoderm, within which are formed muscles, bones, the urogenital tract, and blood; and the ectoderm, responsible for the development of nervous system and epidermal tissue generation. Thus, in contrast to multipotent cells, which form only limited number of cell lineages within the three embryonic layers, pluripotent cells are able to differentiate into any mature cell type. This feature allows a real opportunity to renew non-functional damaged tissues caused by common diseases, such as spinal cord injury, Parkinson’s and Huntington’s diseases, type 1 diabetes, cardiovascular and haematological diseases, osteoarthritis, kidney failure, and stroke (Badger et al. 2014; Bauwens et al. 2011; Blin et al. 2010; Iglesias-García et al. 2013; Kawamura et al. 2012; Kaye and Finkbeiner 2013; Meissner and Jaenisch 2006; Okano et al. 2013; Singla et al. 2011; Thatava et al. 2013; Zhang et al. 2014).

Stem cells occur also in non-embryonic tissues of adult organisms. Precursors of stem cells may spontaneously repair damaged tissues and organs such as bone marrow, gut, and liver. They reside in a special microenvironment called the stem cell niche. Some of these can differentiate into other cell type lineages (Jopling et al. 2011; Li et al. 2005).

Currently, some non-embryonic stem cell (non-ESC) transplantations have been implemented in medical practice, especially in haematology associated with malignancy therapy. Human hematopoietic cells derived from bone marrow, peripheral blood or umbilical cords help to treat individuals undergoing cancer treatment. Chemotherapy-induced bone marrow ablation can be replaced with other bone marrow transplanted stem cells (Copelan 2006).

Unfortunately, adult stem cells (ASCs) are relatively rare and in the bone marrow, hematopoietic stem cells are estimated to be less than 1 in 10,000 cells (Domen et al. 2006). Additionally, non-ESCs have some limitations for application in cellular therapies. A low capacity to self-renew in laboratory conditions reduces the possibility of their efficient multiplication.

Such obstacles prompted scientists to reprogram somatic cells into pluripotent cells in individual patients. Until recently, somatic cell nuclear transfer (SCNT) was the primary technique for producing genetically matched cells and tissues that may potentially cure the diseases of civilization (Gurdon 1962a). The procedure is based on transferring a nucleus from a somatic cell into the enucleated egg. Despite the great progress made in SCNT research in the mouse model, many difficulties appearing in primate cloning have to be overcome to introduce therapeutic applications in humans (Byrne et al. 2007).

Another approach bypassing the use of embryonic cells was a transfection procedure forcing the expression of *Oct4*, *Sox2*, *Klf4* and *c*-*Myc* genes, reported by Takahashi and Yamanaka (2006). They induced pluripotent stem cells (iPSCs) from mouse fibroblasts. One year later the experiment succeeded with human cells (Takahashi et al. 2007). This technique opened a new field of stem cell research for the generation of PSC lines that can be genetically customized for the patient, thus lowering the immune rejection risk. Currently, studies have aimed to implement the technique while excluding carcinogenesis and boosting the reprogramming efficiency by improving transduction systems to augment pluripotency potential.

These and other studies have led to a better understanding of the mechanism of pluripotency maintenance. In addition to the pluripotency genes, three principal cytokines are significantly involved in this process: fibroblast growth factor (FGF)-2, transforming growth factor (TGF)-β/bone morphogenic protein (BMP; especially activin-A) and Wingless-related integration site family of proteins (WNTs) (Sato et al. 2003). Otherwise, critical homologous gene sets responsible for stemness are induced in pluripotent cells, such as the *POU* domain, class 5 transcription factor 1 (*OCT4*), the *NANOG* homeobox, and the sex determining region Y-box 2 (*SOX2*) (Abeyta et al. 2004). These gene sets work within an autoregulatory loop which activates other pluripotency-linked genes (their number is estimated as approximately 350 genes) and repress differentiation-related genes (Boyer et al. 2005). A negative feedback with *Foxd3* ensures stable *Oct4* expression and maintenance of pluripotency (Pan et al. 2006).

## Direct PSC Sampling

PSCs can be obtained from a fertilized embryo growing in vitro for 5 days, the human early stage blastocyst (Thomson et al. 1998). At this stage, the structure consists of the trophoblast, forming the placenta, and the blastocoele, a fluid filling the cavity, and the inner cell mass (ICM), which gives a rise to a foetus. If there were not ethical concerns, this could provide a theoretically unlimited supply of PSCs. Currently, there are six primary approaches to establish human PSC lines from embryonic or foetal tissues, as follows.

- Traditional human ESC (hESC) line generation (embryonic derivative): the first hESC line was generated by Thomson et al. (1998) using ICM from spare in vitro fertilized embryos at the blastocyst phase. ICM cells are pluripotent, with the ability to become any type of cell other than the umbilical cord and the placenta. Following dissection or immunosurgery, ICMs are plated onto an irradiated mouse fibroblast feeder layer and cultured in high serum-containing growth factors medium (Thomson et al. 1998).
- Human primordial germ cells: Gearhart and co-workers isolated primordial germ cells from a 5- to 7-week-old embryo and established embryonic germ cell lines (Shamblott et al. 1998). The issue with this technique was spontaneous undirected cell differentiation.
- ESCs from dead embryos: this technique uses embryos that stopped dividing after in vitro fertilization (Zhang et al. 2006).
- hESC derivatives from genetically abnormal embryos: embryos with diagnosed genetic disorders were employed to obtain hESC lines to understand the mechanism of disorders such as Huntington’s disease, Marfan syndrome, muscular dystrophy and thalassemia (Verlinsky et al. 2005).
- Single cell embryo biopsy as hESC line source: this technique exploits single cells from pre-implanted human embryos without affecting blastocyst viability (Chung et al. 2006).
- hESC generation via parthenogenesis: in this study, a human embryo was generated without fertilization by sperm. The egg was physically triggered to mimic a fertilization event and subsequently divided and formed a blastocyst (Revazova et al. 2007). In this case, pluripotent human stem cells bear genetic information only of egg origin.

All of these methods involve the isolation of pluripotent cells at an early developmental phase followed by culturing cells in vitro. Growing hESCs in culture and maintaining them in a self-renewal and pluripotent state with a stable karyotype is difficult and requires highly specialized techniques. It is frequently supported by mouse or human feeder layers (Richards et al. 2002) or conditioned media in feeder-free culture systems (Xu et al. 2001). Inactivated (stopped cell divisions) feeder layer cells secrete necessary nutrients and proteins promoting growth and detoxifying the culture medium, inter alia, FGF-2 and BMP inhibitor (Dahl et al. 2003; Xu et al. 2005). Fibroblast feeder cells also express adhesion molecules and extracellular matrices, promoting the attachment of pluripotent cells. However, there can be contamination during the later differentiation process (e.g., cross-transferring xenogeneic factors). Thus, these cells must be subsequently removed or replaced with matrix proteins; e.g., Matrigel, laminin (Xu et al. 2001) or synthetic polymers (Mei et al. 2010). This is also the reason for exchanging culture media with defined media; e.g., knockout serum replacement medium, mTeSR1 medium and xeno-free media such as NutriStem (Sugii et al. 2010), TeSR2 (Meng et al. 2012), Essential 8 (Chen et al. 2011) and StemFit (Nakagawa et al. 2014). These are important media for future clinical therapy because animal-derived products may introduce exogenous antigens, pathogens and viruses to the cell population.

The techniques described above involve the destruction of embryos during in vitro fertilization procedures, which prevents wider deployment. However, it is possible to derive new hESC lines using only a single blastomere by culturing the embryo with a mixture of human laminin LN-521, an adhesion protein occurring in ICM, and E-CADHERIN, providing intercellular contact triggering signals (Hovatta et al. 2014). Family specific lines provide advantages requiring a lower dose of immunosuppressants and excluding the extensive formation of banks of haplotypes that represent most human leukocyte antigen histocompatibility classes and ensure the generation of many cell types for cell therapy purposes (Jacquet et al. 2013).

Nevertheless, many ethical concerns related to embryonic cells prompted scientists to investigate new directions in stem cell generation. In 2003, there was some interest in the *OCT*-*4* positive cells from the amniotic fluid that surrounds the foetus (Prusa et al. 2003). It was possible to differentiate them into three embryonic germ layers, and after transferring them into an immunocompromised animal model, no tumor formation was observed. Initial differentiation attempts resulted in the generation of human neuronal, liver and bone cells (Hauser et al. 2010). This research initiated the non-ESC line known as amniotic fluid-derived stem cells (AFS). However, AFSs did not produce all proteins typical for PSCs.

## Somatic Cell Nuclear Transfer

Ethical controversies and a shortage of ASCs forced scientists to search for alternative sources of PSCs. One of the options was “reprogramming” somatic cells, which can be accomplished with SCNT to an enucleated egg.

The idea of nuclear transfer first appeared at the end of 19th century. The first experiments were conducted on fish and amphibian eggs because of their large size and unlimited resources (Spemann 1938). As early as 1962, John Gurdon’s studies on the vertebrate somatic cell nucleus suggested the possibility of “reprogramming” somatic cells into an embryonic state. He transferred a nucleus from a differentiated intestinal epithelial cell into an enucleated egg of *Xenopus laevis*. This technique successfully formed a fertile adult frog (Gurdon 1962b). A few decades later, this method was successfully used for mammalian somatic cell nucleus transfer by Ian Wilmut and Keith Campbell (Wilmut et al. 1997). To date, there have been approximately 15 mammalian species cloned, including the mouse (Wakayama et al. 1998), pig (Polejaeva et al. 2000), rabbit (Chesné et al. 2002), dog (Lee et al. 2005), buffalo (Shi et al. 2007) and camel (Wani et al. 2010). Issues that emerged during the procedure were connected with developmental arrest soon after implantation, abnormal gene expression, incomplete genetic reprogramming and a low success rate, below 5 % regardless of the species used (Wakayama 2007). In addition, cloned animals were affected with frequent pathologies of the brain and kidney, diabetes, obesity, and large offspring syndrome, for example.

To obtain human stem cells via SCNT, human oocytes can be derived from a donor after stimulation with gonadotropins to produce a range of oocytes during the hyperovulation process. The nucleus from the oocyte is then removed and replaced with a nucleus from a somatic differentiated cell of another person. Next, the egg is stimulated by electrical impulses to divide to the blastocyst stage. Then, cells of the inner cell mass are extracted and used to start cultures of ESCs. The procedure can also be performed with nuclei at the blastocyst stage, as reported for two primate species, macaques (Byrne et al. 2007) and humans (French et al. 2008). Somatic cell nuclear transfer was performed with fibroblast nuclei and cultured up to the blastocyst stage. To date, much progress in ESC research has been achieved in the mouse model, whereas in humans, unexpected difficulties appeared concerning reprogramming and the epigenetic status of somatic cell nuclei (Yu et al. 2009b). The first attempts using this technique in humans were carried out by American Cell Technologies in 1998 using a human skin cell and an enucleated egg from a cow. The hybrid human clone developed an embryo that was destroyed after 12 days. Lately, a wide range of cell types, derivatives of all the three embryonic germ layers, have been differentiated from ESCs (obtained by SCNT); e.g., neural cells (Barberi et al. 2003) and/or cardiomyocytes (Lü et al. 2008).

The factors responsible for nuclear reprogramming are found primarily in the oocyte cytoplasm. DNA methylation and histone modification play a paramount role influencing genomic imprinting, X chromosome inactivation, cell differentiation, chromosome structure, cell senescence and apoptosis (Bird 2002). DNA methylation in SCNT embryos is at much higher level than in normal embryos. This may be the reason why demethylation and *de novo* methylation in the somatic cell donor nucleus is not as efficient, resulting often in incomplete reprogramming. Moreover, the somatic nucleus is subjected to histone reacetylation. The epigenetic marks of histones must be erased and reestablished before embryonic transfer to the recipient mother. Due to hyperacetylation in the somatic nucleus, aberrant histone modifications may occur in cloned embryos (Wang et al. 2007). Hence, to obtain the totipotent state, inhibitors of histone deacetylase (HDAC) have been used, such as trichostatin A. Simultaneously, they improved nuclear remodelling by establishing and maintaining a zygotic-like chromatin structure (Maalouf et al. 2009).

A variation of the classical SCNT has been to turn off the *Cdx2* gene (required for implantation in the uterus). The procedure of patient-specific stem cell development is called altered nuclear transfer and does not entail the destruction of the embryo. This technique has been used only in mice (Meissner and Jaenisch 2006) and is highly controversial for use in humans. Moreover, PSCs were successfully generated from the late epiblast of post-implementation embryos, called epiblast stem cells (EpiSCs), with the same efficiency as blastocyst-mediated ICM (Brambrink et al. 2006). However, there were some differences in gene expression and epigenetic patterns between EpiSCs and epiblast cells taken from naturally fertilized embryos (Ding et al. 2009).

Another approach is cell fusion of adult human skin cells with human ESCs in a milieu of some reprogramming factors, as undertaken by Cowan et al. (2005). “Hybrid” cells resembled ESCs in terms of growth and division as well as the expression of proteins typical for pluripotent cells. One crucial obstacle is a problem for further clinical use; the fused cells were tetraploids, with four copies of the DNA in the nucleus. To date, the problem of extra DNA removal has been unsolved. The method is now useful in studying the course of reprogramming adult somatic cells.

The main advantage of SCNT over ESCs (embryo-originated) is the presence of identical genes in the donor and the pluripotent cells derived from its nucleus, which lays a foundation for therapeutic cloning. The goal is based on generating healthy tissues and organs to transplant them into the patient/donor of the nucleus from his/her somatic cells. This method may exclude the problem of immune rejection and organ donation. Some experiments in a mouse model supported the developmental potential of ESCs generated by SCNT and their resemblance to ESCs received from fertilized embryos in respect to DNA microarray profiles, DNA methylated regions, gene-expression and transcriptional profiling, microRNA and protein expression (Brambrink et al. 2006; Ding et al. 2009). Furthermore, compared to iPSCs, no effect of epigenetic memory of the donor cell type was reported in SCNT-derived pluripotent cells, and fewer CpG sites in the promoters retain methylation from parental cells (Ma et al. 2014). These cells may have therapeutic potential also in tissue engineering, when they proliferate and differentiate on scaffold bases. Tissue engineering trials were initialized with myocardial tissue in a rat post-infarction model. The method may also enable the treatment of atherosclerosis, severe skin burns, diabetes mellitus and strokes. Moreover, it was shown that SCNT-derived ESCs can help to correct gene defects (Rideout et al. 2002) and complement neurons in Parkinsonian mice (Tabar et al. 2008).

SCNT may also be useful to create a model for human genetic diseases like Huntington’s disease by using the cell lines for testing drugs and studying cell growth and metabolism. However, the generation of human ESCs by SCNT using aged nuclear donors is still problematic. Nevertheless, introducing transgene encoding telomerase activity could restore telomere length and improve cell survival. Recently, Chung et al. (2014), using Tachibana’s protocol (Tachibana et al. 2013), obtained SCNT-hESCs taking the nuclei from dermal fibroblasts of 35- and 75-year-old males and demonstrated that the nuclear reprogramming of human cells is possible despite age-associated changes. SCNT-derived cells can be used in cancer diagnosis by checking carcinogenesis risk based on genetic or epigenetic defects (Hochedlinger et al. 2004; Li et al. 2003; Novak 2004). However, the epigenetic resetting connected with SCNT hinders proper disease representation (Jaenisch 2004).

Some issues remain unsolved. Cell availability limits clinical application, and the cells are not fully immune compatible (Mombaerts 2003). In addition, the donor egg contains DNA located in the mitochondria, which excludes complete DNA identity to the donor nucleus. And finally, social resistance to human oocyte donation and a lack of clear boundaries between therapeutic and reproductive cloning are large issues.

## Reprogramming Somatic Cells with Gene Overexpression

The milestone in cell reprogramming was to generate iPSCs without using embryos. Takahashi and Yamanaka (2006) introduced 4 selected genes whose overexpression was critical for pluripotency status (*Oct4*, *Sox2*, *Klf4* and *c*-*Myc)* to mouse embryonic fibroblasts (MEFs) and adult tail-tip fibroblasts. The iPSCs from adult somatic cells were similar to ESCs in terms of their morphology, proliferation, pluripotent gene expression, epigenetic patterns, surface antigens and telomerase activity.

These transcription factors change the epigenetic status typical of differentiated cells by silencing retroviral transgenes; reactivating endogenous pluripotency genes; the formation of bivalent chromatin domains in the promoters of developmental genes; DNA hypo- and/or hyper-methylation in imprinted gene *loci*; chromatin fibre reorganization; and, possibly, reactivation of the inactive X chromosome in female iPSCs (Li et al. 2014). *OCT4* and *SOX2* are crucial to maintain pluripotency (Boyer et al. 2005; Loh et al. 2006), while *KLF4* and *c*-*MYC* are proto-oncogenic factors. In addition, *c*-*MYC* induces the global acetylation of histone. High-density oligonucleotide arrays conducted on human chromosomes 21 and 22 revealed as many as 25,000 transcription factor binding site regions for this “master regulator” (Cawley et al. 2004). Thus, *c*-*MYC* largely promotes *OCT4* and *SOX2* binding to specific DNA fragments (Fernandez et al. 2003). *KLF4* is thought to be involved in the inhibition of p53 protein, which downregulates *NANOG* expression during ESC differentiation (Rowland et al. 2005). Further studies have shown that *NANOG* is one of the most important pluripotency factors and the gene set discovered by Takahashi and Yamanaka (2006) may be even reduced to *OCT4* overexpression with extra molecule supplementation for successful pluripotency.

Somatic cells can be reprogrammed (both X chromosomes are inactivated) and followed by injection in the blastocyst they acquire the ability to differentiate into various cell types and tissues that are derivatives of the three embryonic germ layers. The second generation of high quality iPSCs (*Nanog*-iPSCs) was selected by construction of a *Nanog*-reporter under puromycin resistance (Okita et al. 2007). This technique resulted in pluripotent cells competent for germline transmission, successfully generating live chimeric mice (Maherali et al. 2008; Wernig et al. 2007).

Thus, a new perspective emerged in 2007 after the successful generation of human PSCs from adult human fibroblasts. The pluripotency status was assessed by detection of the surface antigen: SSEA-3, SSEA-4, tumor related antigens TRA-1-60, TRA-1-81 and expression of *NANOG*. Other pluripotent properties included high telomerase activity, high pluripotency potential towards the cells originating from three embryonic layers and the generation of teratomas in immunocompromised animals (Takahashi et al. 2007). The technique has been considered to be much easier than SCNT and circumvents ethical controversies. Human iPSCs can be patient-specific, which presents the possibility to screen new drugs in a close-fitting toxicity model. Direct testing of the pluripotency of iPSCs in humans is not possible because of the risk of tumors, which is one of the reasons for the cell therapy delay in clinical trials.

Although successes in embryonic stem cell isolation and iPSC generation have been reported, differences between iPSCs and hESCs still occur. In comparison to ESCs, iPSCs exhibit lower developmental potential and differentiation capacity, depending on various initial states of pluripotency, different conditions of cell line maintenance, epigenetic status originating from the tissue source (Kim et al. 2010), and the distinct ability for the production of intracellular growth factors. Hu et al. (2010) demonstrated that iPSCs differentiate to human neurons with significantly lower efficiency than ESCs, which may be the effect of specific somatic cell genetic reprogramming. Another study comparing ESCs and iPSCs with identical DNA showed a distinct efficiency at incorporation into chimeric mice and remarkably different gene activity on chromosome 12. Insufficient knowledge related to the molecular basis of iPSC generation is one of the drawbacks of this technique. One example is the immunogenic reaction developed against some tissues derived from iPSCs, stemming from unknown genetic and epigenetic defects (Cao et al. 2014).

### Chemically Induced PSCs

The latest development in the field of somatic cell reprogramming is a transgene-free approach. iPSCs may be introduced to clinical trials after disposing exogenous reprogramming factors. With time, studies of genetic reprogramming allowed a gradual reduction in the number of pluripotency genes to be introduced to somatic cells. The *Oct4* transcription factor alone was sufficient when adding the compound “VC6T” (valproic acid, CHIR99021, 616452 and tranylcypromine) to generate iPSCs in murine and human cells (Li et al. 2011; Zhu et al. 2010).

Hou et al. (2013) reported that after screening 10,000 small molecules, forskolin (cAMP agonist), 2-MeHT (2-methyl-5-hydroxytryptamine) and D4476 (casein kinase inhibitor) could also be a chemical substitute for *Oct4*. The expression of *Oct4* and *Nanog* was not observed. Moreover, their promoters were hypermethylated. For successful reprogramming, the composition of additional molecules was further required.

Optimization of the procedure with VC6T, DZNep (3-Deazaneplanocin A), VC6TF (VC6T plus forskolin), glycogen synthase kinase-3 and mitogen-activated protein kinases was attempted. Cells termed chemically iPSCs (CiPSCs), of ESC-like morphology with compatible gene expression profile that differentiate into three germ layers, were generated. The efficacy of this experimental process was up to 0.2 % (comparable to reprogramming obtained by classical transcription factors); that is, the generation of 1–25 CiPSC colonies from 50,000 plated MEFs. After injection into the blastocyst, integration with the other organs was detected, including the gonads, as well as transmission to the following generation. Moreover, the chimeric mice generated from these cells were completely viable and healthy, evidence of a full reprogramming process (Hou et al. 2013).

Further research established several essential compounds for pluripotency induction: C (CHIR99021—a glycogen synthase kinase 3 inhibitor), 6 (616452—TGF-β inhibitor), F (forskolin) and Z (DZNep—a S-adenosyl homocysteine hydrolase inhibitor; an epigenetic modulator), and compound P (PD0325901), responsible for the pluripotency circuit. In addition, optional “minimal essential” booster compounds were identified: V (VPA), T (Tranylcypromine, Pernate) and T (TTNPB—a synthetic retinoic acid receptor ligand). CF6 molecules are thought to induce an early phase of *Sall4* and *Sox2* overexpression, DZNep, for the *Oct4* gene by decreasing DNA methylation at the late step of the chemical reprogramming stage. Subsequently, these molecules activate genes associated with pluripotency, for example the *Nanog* gene, and silence *Gata6* (Masuda et al. 2013). As a result, CiPSCs demonstrate doubling time, alkaline phosphatase activity, DNA methylation status and pluripotency markers with normal karyotype and genetic integrity up to 13 passages, similar to ESCs.

The CiPSC technique was successfully used in mouse adult fibroblasts, mouse neonatal fibroblasts and adipose-derived stem cells but not in human cells. However, the use of this technique in humans seems to be only a matter of time, although it probably will require some modifications.

## Differences between Mice and Human Pluripotency

Pluripotency can be considered in two states. Naïve pluripotent cells (mouse ESCs) were first obtained from ICM cells derived from preimplantation mouse embryos in vitro and were similar to ICM cells at embryonic day 4.5, capable of giving rise to chimeric animals with high yield and insensitive to single cell dissociation. In female mice, both X chromosomes were active (De Los Angeles et al. 2012). The second (more rare) subpopulation is known as primed PSCs, consisting of more developed cells. This subpopulation originates from mouse EpiSCs biopsied from early post-implantation embryos. In spite of creating chimeras, in females only one chromosome X was active and sensitivity to single cell dissociation occurred. Differences were also noted in flatter colony morphology (Huang et al. 2012). Human ESCs, although derived from ICM, resembled mouse EpiSCs in terms of required growth factors and X chromosome activity (De Los Angeles et al. 2012). Nevertheless, Gafni et al. (2013) managed to derive human naïve PSCs with similar features to mice counterparts. However, comparative computational analysis revealed their similarity to the primed mice PSC state, in respect to the cellular and differentiation-related response (Ernst et al. 2015).

Research into the mechanisms regulating pluripotency was conducted largely based on mouse iPSCs. Subsequently, some differences were found in pluripotent cells originating from human and mice PSC lines (Schnerch et al. 2010). Even before developing the protocol for obtaining iPSCs across species, some disparities in establishing ESC lines were determined, including distinct development, timing of isolation and global transcriptional profile (Rao 2004; Sato et al. 2003). Morphologically, human embryonic stem colonies do not override each other and are flatter. As with iPSC culture, they require FGF-2 for self-renewal rather than leukemia inhibitory factor in mice pluripotent cells (Amit et al. 2000; Matsuda et al. 1999). In turn, BMP, promoting self-renewal in mouse ESCs, causes differentiation in human PSCs (Xu et al. 2005; Ying et al. 2003). Research suggests that the main transcriptional network controlling pluripotency in mice and humans is consistent. Detailed differences lie in distinct signals and external factors maintaining self-renewal (Yu et al. 2007), as well as in their targets governing cell fate; i.e., overexpression of the *c*-*MYC* factor resulting in differentiation and apoptosis of human ESCs but not murine ESCs (Cartwright et al. 2005).

Some differences were also apparent during iPSC generation that can be explained by slower doubling time and silencing kinetics of ectopically expressed genes in human PSCs (Koch et al. 2006), which may have an impact on lower production yield (Masaki et al. 2007). Although *Oct4*, the master regulator of pluripotency, is essential for reprogramming in mice and human PSCs, the effects of gene knockout or increasing the level of *Oct4* caused spontaneous differentiation in a diverse manner for these two species (Niwa et al. 2000; Rodriguez et al. 2007). The functionality of *Sox2* is much better known for mouse iPSCs than for human pluripotent cells. In turn, the induction of different differentiation pathways between human and mice ESCs was shown in the case of *Nanog* downregulation (Hyslop et al. 2005; Mitsui et al. 2003; Zaehres et al. 2005). In comparison with human PSCs, Kruppel-like factor (*Klf*) family members are upregulated in mouse iPSCs, resulting in differences in self-renewal regulation (Jiang et al. 2008). Additionally, the impact of miRNAs on regulating pluripotency, including occupying the promoters of *Oct4*, *Sox2* and *Nanog,* is not without significance. Interspecies distinctions in miRNA profiles and their distribution in chromosomes may be reflected by cell differentiation. The same applies to some genes that may be not epigenetically conserved within particular mammalian species (Ivanova et al. 2006). Also there are some species-specific markers; e.g., SSEA-1 occurs in mice but not in humans. All these differences suggest that studies performed using mice cell lines cannot be directly translated to the biology of human pluripotent cells. Thus, the mechanism of differentiation and its regulatory pathways in human iPSCs must be more deeply understood for wider applications of stem cell replacement therapy. Finally, designing new functional assays for human iPSCs could replace currently used animal-based models and single cell assays.

## Pluripotency Assessment

The key issue for conducting research on pure pluripotent cell generation is assessing the quality of the method. Investigating the scope of cell pluripotency is a fundamental stage prior to clinical applications (Table 1). A normal phenotype initially allows for consideration of a particular cell line to undergo the differentiation procedure and later to be transplanted into the patient. One of the first indicators is a high nuclear to cytoplasmic ratio. Moreover, undifferentiated hESCs form compact multilayer colonies with defined edges and high alkaline phosphatase activity (Reubinoff et al. 2000). Cell immortality is connected to a high level of telomerase activity (Thomson et al. 1998). Pluripotency is also characterized by examining the level of alkaline phosphatase. Finally, many specific epitopes and nuclear transcription factors associated with pluripotency can be recognized, proving its genetic function and differentiation potential (Table 2).

**Table 1:** Main assays for pluripotency characterization according to their priority

Pluripotency characterization	Significance	Importance	Drawbacks
Alkaline phosphatase activity	Placental AP is upregulated in PSCs	– AP staining is used for early screening (initial indicator of successful reprogramming) and distinguishing PSCs from feeder and parental cells – May serve as a reporter of transfected cells after thermal treatment	– Extra pluripotent tests needed – Some reagents toxic for PCSs result in loss of cellular morphology and preclude further propagation – Level of AP expression depends on cell line – Background noise (signals)
Stem cell surface marker detection (immunostaining)	The keratan sulfate antigens TRA-1-60, TRA-1-81 and the glycolipid antigens SSEA3 and SSEA4 determine pluripotent cells	– Phenotypic assessment of the pluripotent status and homogeneity in cell culture – Valuable time– saving and easy– to– handle method to distinguish, select and isolate reprogrammed colonies	– required “negative control” to suppress a background signal – Incomplete qualitative confirmation of pluripotency at the protein level
Pluripotent marker expression (RT-PCR and protein assays)	Detection of the set of pluripotent genes and proteins	– Quantitative analysis of undifferentiated marker gene expression and efficacy confirmation of transgene silencing by RT-PCR – Complementary detection of pluripotent nuclear protein markers	– Assays do not fully confirm the complete reprogramming process – Positive results do not prejudge the pluripotent abilities in in vivo conditions
Epigenetic status of promoter	Indicator of gene expression related to pluripotency	– Evaluation of the methylation status of CpG in pluripotency-associated gene promoters (bisulphite sequencing) and epigenetic status of H3 histones (e.g. by chromatin immunoprecipitation) in comparison to PSCs and parental cells verifies pluripotent status of cells and allows to check whether epigenetic memory remained from somatic cells and PSCs is safe and effective for clinical application – Level of transcriptional activity may be detected via luciferase reporter assay – Global DNA methylations ensures to assess the reprogramming progress	– The analysis alone is not sufficient to conclude pluripotency – Multistep and complex test to conduct – The role of epigenetic regulation of gene expression not fully understood
Gene expression profiling (DNA microarray)	Global gene expression patterns as a relative for ES and parental cell expression profile	– Straightforward and relatively inexpensive method for verification of differences between pluripotency status of iPSCs, ESCs and descendant cell lines – Convincing prove for complete reprogramming process	– Positive results do not prejudge the pluripotent abilities in in vivo conditions
Differentiation tests in vitro (EBs)	Ability of PSCs for forming tissues derived from the three primordial germ layers	– Quick and easy-to-handle for demonstration of pluripotent abilities of PSC lines – Immunohistochemistry assays detect three germ layer markers	– Positive results do not prejudge the pluripotent abilities in in vivo conditions – Still recommended direct in vitro differentiation
Direct in vitro differentiation	Assessment of pluripotency towards specific cellular lineage	– Ability to differentiate into specific tissue confirmed by immunostaining and RT-PCR analysis	– Does not prove full pluripotency of particular iPS cell line
Differentiation tests in vivo (teratoma)	The most rigorous and definite landmark test for pluripotency	– Easy to obtain, no problematic procedure of cell administration into animal – Immunohistochemistry and histopathological analysis detects three derivation tissues of germ layers – Spontaneous teratoma formation may help in understanding potential hazards of stem cell therapies	– Poor reproducibility and high degree of variability – Time-consuming, laborious procedure – Teratoma assay requires a threshold level of cells to prove successful reprogramming – Not standardized in terms of graft size, number of implanted cells, mice age, PSC preparation

The tests presented in the table are directly related to assessing pluripotency of PSC lines—gene expression profiling, epigenetic status (promoter), teratoma, formation and in vitro cell differentiation. However, additional assays connected with utility in further application studies are highly recommended, namely telomerase activity (TERT expression is responsible for self-renewal abilities of PSCs), doubling time (for comparing further rate of cell divisions between iPSCs, ESCs and descendant cell lines), cross-contamination test (DNA fingerprinting like STR analysis confirms the origin from parental cells), karyotyping (chromosomal G-band analysis enables detection of chromosomal abnormalities but does not detect minor genetic variations; FISH analysis indicates chromosomal translocation or deletion), genome-wide single-nucleotide polymorphism array analysis (detects the DNA mutation ratio)

*AP* alkaline phosphatase; *EB* embryoid body; *PSC* pluripotent stem cell; *SSEA3* stage specific embryonic antigen-3; *SSEA4* stage specific embryonic antigen-4; *TRA-1-60* tissue rejection antigen 1–60; *TRA-1-81* tissue rejection antigen 1–81

**Table 2:** Main markers of human pluripotency

Stem cell genes		Stem cell surface marker		Signal pathway-related intracellular marker	
Name	Reference number	Name	Reference number	Name	Reference number
Octamer-binding transcription factor 4 (*OCT*-*3/4*, also called *POU5F*)	NP_002692	Stage specific embryonic antigen-3 (SSEA-3)		SMAD family member 2 (SMAD2)	NP_034884
Sex determining region Y-box2 (*SOX2*)	NP_003097	Stage specific embryonic antigen-4 (SSEA-4)		SMAD family member 3 (SMAD3)	NP_005893
Kruppel-like factor 4 (*KLF4*)	NP_004226	Stage specific embryonic antigen-5 (SSEA-5)		SMAD family member 4 (SMAD4)	NP_005350
NANOG homeobox (*NANOG*)	NP_079141	Tissue rejection antigen 1-60 (TRA1-60)		Cadherin-associated protein), beta 1 (β-catenin)	NP_001091680
		Tissue rejection antigen 1-81 (TRA1-81)			
Growth differentiation factor 3 (*GDF3*)	NP_065685	Tissue rejection antigens 2-49/54 (TRA2-49/54) detecting alkaline phosphatase (AP)			
Fibroblast growth factor-4 (*FGF4*)	NP_001998	L1 cell adhesion molecule (L1CAM)	NP_000416		
Undifferentiated embryonic cell transcription factor 1 (*UTF1*)	NP_003568	E-cadherin type 1 (epithelial) (CDH1, CD324)	NP_004351		
Human telomerase reverse transcriptase (*hTERT*)	NP_937983	Thymus cell antigen 1 (CD90)	NP_006279		
Reduced expression 1 (*REX1*, *ZFP42*)	NP_065746	c-KIT (CD117, SCFR)	NP_000213		
Developmental pluripotency associated 2 (*DPPA2*, *ECSA*)	NP_620170	β1 integrin (CD29)	NP_002202		
Developmental pluripotency associated 5 (*DPPA5*, *ESG1*)	NP_001020461	Platelet/endothelial cell adhesion molecule 1 (PECAM-1, CD31)	NP_000433		
Telomeric repeat binding factor (NIMA-interacting) 1 (*TERF1*)	NP_059523	Frizzled5 (FZD5)	NP_003459		
Sal-like 1 (Drosophila) (*SALL1*)	NP_001121364	Teratocarcinoma-derived growth factor 1 (TDGF1, Cripto)	NP_003203		
Lin-28 homolog A (C. elegans) (*LIN28A*)	NP_078950	Lectins, receptor binding short peptides			
Zic family member 3 (*ZIC3*)	NP_003404				
DNA (cytosine-5-)-methyltransferase 3 beta (*DNMT3B*)	NP_008823				
NODAL modulator 2 (*NOMO2*)	NP_001004060				
Orthodenticle homeobox 2 (*OTX2*)	NP_068374				
Left–right determination factor 1 (*LEFTY1*)	NP_066277				
Forkhead box D3 (*FOXD3*)	NP_036315				
Signal transducer and activator of transcription 3 (*STAT3*)	NP_644805				
Zinc finger protein X-linked (*ZFX*)	NP_001171555				

The ability to form three embryonic germ layers can be determined in vitro by culturing ESCs in suspension to form embryoid bodies, cell aggregates which contain derivatives of the mesoderm, endoderm and ectoderm (Zeng et al. 2004). Analogous differentiation processes also occur after PSCs implantation into a host animal, usually a mouse. The standard pluripotency assay in vivo is based on the generation of teratomas in SCID mice. The heterogeneous structures are basically encapsulated tumors made up from cells at varying differentiation stages and of different embryonic layer origin. Next, the teratomas are evaluated for histopathology (Steiner et al. 2010). This method, however, is subjected to poor reproducibility and a high degree of variability.

Under more advanced verification, human ESCs are subjected to direct differentiation into desired cellular lineages. This homogenous population serves as an indicator required for further cell transplantation consideration. It is a proof of the pluripotency and differential flexibility of a particular ESC line capable of forming different tissues. Neural differentiation protocols are used to show ectoderm direction (Nat et al. 2007), hepatocyte differentiation for endoderm derivatives (Cai et al. 2007) and cartilage or cardiac differentiation protocol for mesodermal fate (Mummery et al. 2007; Qu et al. 2013).

A widely used easy and quick immunostaining method for fixed pluripotent cells shows homogeneity in monolayer culture. Nevertheless, it is a qualitative assay and it must be used with a “negative control” to ensure background signal suppression. Therefore, it must be carried out alongside quantitative methods, as it is confirmed by images of embryoid bodies disclosing pluripotency markers even after a long period of partial cell differentiation (Bhattacharya et al. 2005). Hence, quantitative reverse transcription-polymerase chain reaction analysis, confirming stable and abundant levels of given marker expression, is a necessary step. To evaluate the three main germline lineages, markers must be appended with a view to the neuro-ectoderm path being the main direction of hESCs differentiation (Vallier et al. 2004). In turn, immunoblots give semi-quantitative data related to protein markers. Fluorescence-activated cell sorting (FACS) is a supplementary test detecting different cellular populations within hESC cultures (Silva and Smith 2008).

Researchers have also raised the relevance of the correct karyotype. Prolonged ESCs culture causes copy number variations and loss of heterozygosity resulting in altered gene expression, precluding its clinical application (Narva et al. 2010). Overall, human ESCs with normal karyotypes generate teratomas composed of only differentiated cells. On the other hand, karyotypically irregular ESCs give rise to teratocarcinomas containing undifferentiated cells, which can indicate genetic abnormalities (Sidhu 2012). This is the reason for the required karyotyping of pluripotent cells designed for cellular therapies after each 10^th^ passage in in vitro conditions. However, in iPSC technology, some iPSC lines with normal karyotypes are subjected to tumorigenic mutations acquired during or after genetic reprogramming. For example, in a small area it may come to multiplication of the *BCL2L1locus* connected with an anti-apoptotic factor (Amps et al. 2011). Thus, for potential clinical use, they should be subjected to extensive genetic screening. It is assumed that for PSC transplantation to the patient, genotyping with a genome-wide single nucleotide polymorphism array including copy-number variations and copy-number neutral loss-of-heterozygosity regions will bring enough resolution to ensure proper verification of genetic integrity, though it cannot replace karyotyping, which additionally detects balanced translocations and inversions.

In animal models, the pluripotency status is determined by a blastocyst complementation assay. In this method, cells are injected into the developing mouse blastocyst. In the differentiating embryo, the formation of tissues originating from all three embryonic germ layers and chimeric mice is expected (Hochedlinger and Jaenisch 2006). Tetraploid blastocyst complementation is a very valuable pluripotency method to assess the degree of resemblance between reprogrammed iPSCs and normal ESCs (Zhao et al. 2010). As reported by Kang et al. (2009), using this technique, iPS cell lines can generate full-term mice giving the most stringent proof of full pluripotency. Tetraploid embryos are generated by applying an electrical current and fusing late two-cell-stage embryos to one-cell-stage embryos collected from impregnated mice. iPSCs are microinjected into the cavity of the tetraploid blastocyst (4N) and transferred to pseudopregnant ICR strain (Institute of Cancer Research) mice. Viable animals form iPSC-complemented ICR mice tetraploid embryos, demonstrating comparable iPSCs to ESCs potential. Given the ethical limitations, the procedure is not used for human iPSCs.

Instead, many tests are conducted to compare iPSCs with human ESCs; i.e., transcriptome, proteome, microRNA content, epigenome and genome-wide CpG methylation profiling cultured cells (Chin et al. 2009; Guenther et al. 2010). In this regard, RNA arrays and mass spectrometry are very powerful assays for detection markers of pluripotency and differentiation. In addition, comparative genomic hybridization arrays can be used to determine genetic stability, while fluorescent in situ hybridization can localize any possible aneuploidies (Jacobs et al. 2014). In the case of specific cells such as PSCs, high-throughput DNA sequencing (ChIP-Seq) provides essential information about chromatin organization and the pattern of histone modifications near *Oct*-*4* and *Nanog* promoters and other critical genetic *loci* (Bibikova et al. 2008).

## Techniques of PSC Derivation and Potential Clinical Application

The greatest barrier for the medical use of embryonically derived or autologous-induced PSCs is the safety of cell transplantation. Another problem is the generation of a sufficient quantity of purified and functionally active differentiated cells. Moreover, there are conserved gene expression networks which are common for tumorigenesis and cells of pluripotent potential (Lee et al. 2013). In fact, PSCs injected subcutaneously form teratoma-like tumors and can extensively proliferate. This is a clear demonstration that PSC-derived differentiated cells must be very pure before transplantation to the patient. In this respect, ESCs can be considered as a better source than iPSCs with transduced additional genes. However, prolonged in vitro culture may lead to undesired changes at the gene or chromosome level (previously described). Hence, many researchers in the stem cell field are now engaged in designing methods for purifying differentiated cells. For this purpose, FACS and magnetic cell sorting are useful tools, which can be further supported by applying cytotoxic antibodies against pluripotency genes (Choo et al. 2008); cell growth specific inhibitors, such as oleate synthesis inhibitor (Ben-David et al. 2013); and inducible factors of apoptosis with iCasp9 (Stasi et al. 2011) to remove all the remaining pluripotent cells.

For ESC derivation, human zygote exploration is impossible to avoid. However, immune rejection is a problem due to the limited number of ESC lines. Matching to highly polymorphic human leukocyte antigens (HLA) is difficult in humans. Still, there are some advantages of ESCs over iPSCs. They seem to have better genetic stability and are more carefully tested, which means that they are safer for cellular therapies. Additionally, they have a much higher ability for expansion and regenerative potential than cord blood and mesenchymal stem cells. Embryonic cells maintain their differentiation potential for 3–6 days after fertilization and can fulfil many goals of regenerative medicine, such as replacing dead or damaged cells/tissues or building human organs, in spite of their allogenic origin.

Clinical-grade ESCs must comply with many requirements to minimize any possible risk for the recipient. They must fulfil good manufacturing practice, preclude tumorigenesis, and be successfully characterized with in vitro and in vivo assays, including those of genetic stability. The first clinical trials of differentiated oligodendrocytes in spinal cord injury (Piltti et al. 2013) and retinal pigment epithelium (RPE) transplanted to patients with Stargardt’s macular dystrophy (Schwartz et al. 2015) reported no side effects.

The primary expectations with respect to iPSCs are related to immune rejection and ethical issues. However, regardless of progress, there are still some hindrances to clinical trials. The primary studies focused on developing appropriate methods to generate sufficient quality iPSCs with respect to the reprogramming factor, the vectors for exogenous gene delivery and the sources of particular cell types (Seki and Fukuda 2015).

The first principle of regenerative medicine is to avoid the risk of tumor formation by reducing the pluripotential gene set, particularly one containing protooncogenes. In fact, it has been demonstrated that only *Oct4* and *Sox2* are strictly required for pluripotency status, even when not adding extra molecules. Research has concentrated on omitting the *c*-*Myc* oncogene (Okita et al. 2008). Substitutes of this gene have been found, including *Tbx3* (Han et al. 2010), *Glis1* (Maekawa et al. 2011) and *Zscan4* (Jiang et al. 2013). Additionally, Montserrat et al. (2013) successfully replaced Yamanaka factors with the cell lineage specifiers *OCT3/4* with *GATA3*, a mesodermal lineage marker, and *SOX2* with ectodermal marker *ZNF521* (Montserrat et al. 2013).

The method of transgene delivery into somatic cells is crucial for the clinical use of iPSCs (Table 3). At first, the principal method of pluripotency induction was to use a gene introduced by viral-origin vectors: retrovirals, such as pMXs (Takahashi et al. 2007) or pMSCV (Hawley et al. 1994), and lentivirals (Table 4). These vectors ensured high reprogramming efficiency (defined as the percentage of GFP-positive iPSC colonies with appropriate morphology, marked with a GFP-labelled transgene). Successful reprogramming was effective in transgene silencing but a certain risk was associated with the possibility of insertional mutations to the genome because there is a chance for such vectors to integrate randomly (to a genome), potentially leading to the development of some disorders bound to viral transgene reactivation. Moreover, pluripotency genes, such as *c*-*MYC* and *KLF4*, classified as protooncogenes, may induce tumor formation (Okita and Yamanaka 2011) and so are not safe to use in cellular therapy. Some approaches have delivered transcription factors under a single promoter in a single virus, thus reducing the chance of a transgene for mutational insertion into the genome (Carey et al. 2009). In spite of genomic integration, commonly used lentiviruses enabled even more efficient transduction than retroviruses (Yu et al. 2007), but were more resistant to silencing transgenes in the pluripotent state, which rules them out from clinical trials. There is an oncogenic risk in differentiated cells, and transgenes may interfere with functional genes. In turn, a Cre-deletable lentivirus system was not fully effective because LoxP sequence breaking genes remained in the genome after cutting off the insert sequence.

**Table 3:** Advantages and disadvantages of methods used for human iPSC derivation

Type of vector	Introduced factors	Advantages	Disadvantages	References
Viral vectors				
Retroviral vector	*OCT3/4, SOX2, KLF4, c*-*MYC, NANOG*	Well established method, low costs, quite high reprogramming yield, reasonable efficiency	Random integration of transgenes, external sequences remain in genome with incomplete silencing and risk of tumorigenesis, potential chance for infecting human cells and carcinogenesis in researchers stemming from propagation proto-oncogenes via amphotropic and pantropic retroviruses	Takahashi et al. (2007), Lowry et al. (2008)
Lentiviral vector	*OCT3/4, SOX2, KLF4, c*-*MYC,UTF1,p53 siRNA, Slc7a1*	Higher efficacy than in retroviral method, transduction to dividing and non-dividing cells, control of factor expression		Takahashi et al. (2007), Zhao et al. (2008)
Adenoviral vector	*OCT3/4, SOX2, KLF4, c*-*MYC*	Transient gene expression, no residual transients	Conceivable integration to the genome, low efficiency	Zhou and Freed (2009)
Sendai vector	*OCT3/4, SOX2, KLF4, c*-*MYC*	No genome integration, higher reprogramming efficiency than by retroviruses, gradually decreasing transgene quantity with cell divisions	Expensive kits, some difficulties in purifying cells with replicating virus, sequence-sensitive RNA replicase	Fusaki et al. (2009)
Non-viral vectors				
Plasmid	*OCT3/4, SOX2, KLF4, L*-*MYC, LIN28, p53 shRNA*	No genomic integration	Very low efficiency, loss of episomal vectors in reprogramming	Okita et al. (2011)
*piggyBac* transposon	*OCT3/4, SOX2, KLF4, c*-*MYC*	No genomic integration, precise transgene deletion, reasonable efficiency	Inefficient integration and work-consuming procedure of excised line screening	Woltjen et al. (2009)
miRNA	miR-200c, miR-302 s, miR-369 s family miRNAs	No genome integration, more effective than vector methods, faster reprogramming kinetics than with viral vectors	Very low efficiency, time-consuming procedure, fast miRNA degradation, complicated miRNA modification	Miyoshi et al. (2011)
Synthetic modified mRNA	*OCT3/4, SOX2, KLF4, c*-*MYC, LIN28*	No genomic integration, higher efficacy than with retroviruses, no immune antiviral response, faster reprogramming kinetics, more controlled efficacy than with retroviruses, no immune antiviral response	Multiple rounds of transfection required	Warren et al. (2010)
Episomal vectors	*OCT4, SOX2, NANOG, KLF4, c*-*MYC, LIN28, SV40LT*	No genomic integration, completely vector and transgene-free iPSC generation	Low efficacy	Yu et al. (2009a)
Artificial chromosome vetors (HACs)	*OCT/4, SOX2, KLF4, c*-*MYC*, p53 shRNA	No genomic integration, vector and transgene-free iPSC generation, homogenous transgenic expression, built-in safeguard system	Low transfer rate of HACs, low efficacy, work and time-consuming procedure	Hiratsuka et al. (2011)
Minicircle DNA	*OCT4, SOX2, LIN28, NANOG*	No genomic integration, drug selection, no vector excision needed, easy to handle, approved by FDA gives potential clinical application	Low efficacy compared to viral methods, longer ectopic expression	Jia et al. (2010)
Protein transduction	OCT3/4, SOX2, KLF4, c-MYC	No genomic integration, direct delivery of transcription factors, no genetic modification, no immune response, good cellular permeability, no issues connected with DNA, inhibited cellular senescence, attractive to clinical trials	High laboratory requirements, protein chemistry equipment, low efficacy, short half-life of proteins, requirements of large quantities of pure proteins, multiple protein application	Zhou et al. (2009)
Small molecules	CHIR, 616452, FSK, DZNep, PD0325901, VPA, Tranylcypromine, TTNPB	No genomic integration, readily accessible, no genetic modification, low compound costs, no immune response, easy to handle - synthesis, preservation, standardization, reversible function, attractive to clinical trials	Not proved in human cells, no recent reports of full cell reprogramming with small compunds only, low efficacy, time-consuming, possible genetic instability	Hou et al. (2013)

It must be noted that introduction of reprogramming factors by its overexpression is associated with the risk of tumorigenicity and other malfunctions, e.g. *c*-*MYC* is a proto-oncogene, a multifunctional transcription factor involved in cell growth control, apoptosis and differentiation (its adverse interfering with human cells may be diminished by introducing defined transgenes in ecotropic retroviruses into human cells which express murine specific retrovirus receptors as *Slc7a1,* it precludes the infection of normal receptor-negative human cells*)*; *hTERT* by lengthening telomeres prevents apoptosis and potentially makes cells immortal; SV40 T antigen: a proto-oncogene is responsible for cell transformation including interfering with tumor suppressor proteins. Moreover, valproic acid as a histone deacetylase (HDAC) inhibitor inducing chromatin remodeling and upregulating pluripotent genes however is known as a reducing agent towards DNA repair mechanisms during cell divisions. Using short hairpin RNA vectors against p53 represses the main tumor suppressor in the cell which results in increasing rates of DNA damage. Also micro RNA technology still requires better understanding of the full scope of selected miRNAs and their targets

616452, transforming growth factor-beta inhibitor; CHIR, a glycogen synthase kinase 3 inhibitor; *c*-*MYC,* Myc proto-oncogene; c-MYC, Myc proto-oncogene protein; DZNep, a S-adenosyl homocysteine (SAH) hydrolase inhibitor; FSKL, forskolin; *hTERT*, human telomerase reverse transcriptase; *KLF4*, Kruppel-like factor 4; KLF4, Kruppel-like factor 4; *LIN28,* Lin-28 homolog A; *NANOG,* NANOG homeobox; *OCT3/4*, octamer-binding transcription factor 4; OCT3/4, octamer-binding transcription factor 4 (protein); PD0325901, mitogen-activated protein kinase inhibitor; shRNA, short hairpin RNA; Slc7a1, solute carrier family 7 (cationic amino acid transporter, y + system), member 1 (mouse ecotropic receptor for retroviruses also known as mCAT1); *SOX2*, sex determining region Y-box2; SOX2, sex determining region Y-box2 (protein); SV40T, Simian vacuolating virus 40 T antigen; TTNPB, retinoic acid analog; *UTF1,* undifferentiated embryonic cell transcription factor 1; VPA, valproic acid

**Table 4:** Methods for human iPSC derivation by viral vectors

Reprogrammed human cells	Vector of transgene delivery	Introduced transgenes	Efficacy (%)*	iPSC charakterization										References
				Pluripotent marker expression	Epigenetic status of promoter	Microarray analysis	Pluripotency tests		Differentiation potential			Karyo-typing	Cross-contamination test	
							EB	Teratoma	Endodermal	Ectodermal	Mesodermal			
Neonatal/adult skin fibroblasts	Lentiviral	*Sl*	0.02	Yes	Yes	Yes	Yes	Yes		Yes	Yes	Yes	Yes	Takahashi et al. (2007)
	Retroviral	*O, S, K, M*												
Neonatal skin fibroblasts		*O, S, K, M, N*	ND	Yes		Yes	Yes		Yes	Yes	Yes	Yes	Yes	Lowry et al. (2008)
Fetal lung fibroblasts		*O, S, K, M*	0.04	Yes	Yes	Yes	Yes					Yes	Yes	Park et al. (2008)
ES-derived embryonic fibroblasts		*O, S, K, M, T, SV*	0.3					Yes			Yes			
		*O, S, K, M*	0.1											
Mesenchymal cells from umbilical cord (UMCs)		*O, S, K, M*	0.4	Yes	Yes	Yes	Yes	Yes		Yes		Yes	Yes	Cai et al. (2010)
Fetal skin fibroblasts	Lentiviral	*O, S, L, N*	0.02	Yes	Yes	Yes	Yes	Yes				Yes	Yes	Yu et al. (2007)
Neonatal/adult skin fibroblasts		*O, S, K, U,* P	0.1	Yes	Yes	Yes	Yes	Yes	Yes	Yes	Yes	Yes	Yes	Zhao et al. (2008)
Neonatal skin fubroblasts	Adenoviral	*Sl*	0.01	Yes	Yes	Yes		Yes				Yes	Yes	Masaki et al. (2008)
	Retroviral	*O, S, K, M*												
Neonatal/adult skin fibroblasts	Sendai virus	*O, S, K, M*	1	Yes	Yes	Yes	Yes	Yes	Yes	Yes	Yes	Yes	Yes	Fusaki et al. (2009)

AA, ascorbic acid; K (*KLF4*), Kruppel-like factor 4; L (*LIN28*), lin-28 homolog A; M (*c*-*MYC*), Myc proto-oncogene protein; N (*NANOG*), *NANOG* homeobox; O (*OCT3/4*), octamer-binding transcription factor 4; P (p53 siRNA), S (*SOX2*), sex determining region Y-box2; SV (SV40T), simian vacuolating virus 40 T antigen; Sl (Slc7a1), solute carrier family 7 (cationic amino acid transporter, y + system); member 1 (mouse ecotropic receptor for retroviruses also known as mCAT1); T (*TERT*), human telomerase reverse transcriptase; U (*UTF1*), undifferentiated embryonic cell transcription factor 1; VPA, valproic acid

* According to Yamanaka’s method, by which the number of cell colonies was counted and divided by the number of cells plated

The alternative option is the replacement of virus-origin vectors with vectors that do not integrate into the genome, like adenoviruses (Zhao et al. 2008) or the widely used Sendai viruses with transient expression (Fusaki et al. 2009). Safer adenovirus vectors provide transient gene expression without transferring residual transgenes (Harui et al. 1999). Still, integration into the genome and low efficiency are problematic. In turn, the Sendai virus improves clinical potential by introducing negative-sense single stranded RNA into the cytoplasm and not crossing into the nucleus, precluding genomic insertion (Fusaki et al. 2009). Expression of the transgene gradually silences, with cell divisions preventing its reactivation. Any traces of viral RNA can be removed through siRNA infection or a higher temperature treatment (Ban et al. 2011; Nishimura et al. 2011).

However, scientists hold their greatest hope for cellular therapies in non-viral integration-free vectors by employing episomal plasmids (Okita et al. 2007); *piggyback* transposons (Woltjen et al. 2009); the most effective EV method (Yu et al. 2009a); human artificial chromosome vectors (Hiratsuka et al. 2011) or minicircle DNAs approved by the FDA (Jia et al. 2010); or proteins, RNAs and small chemical molecules (Rajasingh 2012). Safety can be achieved at the expense of decreased efficiency and longer pluripotency activation; however, all these approaches tend to apply to iPSCs for prospective clinical use.

Refined protocols with episomal vectors bearing combinations of *OCT3/4*, *SOX2*, *KLF4*, *L*-*MYC*, *LIN28*, and the shRNA for the TP53 plasmid brought higher virus-free pluripotency induction than previously (Okita et al. 2011, 2013). Another approach to eliminate residual transgenes is the piggyBac transposon system based on piggyBac transposase. Integration-free iPSCs were an advantage but reprogramming efficiency needs to be improved (Woltjen et al. 2009).

There are promising methods related to RNA-based reprogramming. The method of direct delivery of synthetically transcribed mRNAs in in vitro conditions precluded endogenous antiviral cell defence and triggered somatic cell reprogramming with higher efficiency compared to retroviruses (Warren et al. 2010). Also, miRNAs as pluripotency carriers are taken into account for potential clinical use (Miyoshi et al. 2011). Specific mature miRNA combinations through lentiviral transduction have the capacity to reprogram somatic cells into iPSCs without exogenous transcription factors. For example, the expression of a *miR302/367* cluster (comprised of 5 miRNAs) by *miR367* activation initiates the process two-fold more effectively than *OCT4/SOX2/KLF4/MYC*-derived methods and precludes genes breaks or transgene reactivation (Anokye-Danso et al. 2011). Simultaneously, linkage with the HDAC-mediated pathway was determined, in which the miRNAs targets are *Oct4* and *Sox2* aimed to maintain cell homeostasis and pluripotency in ESCs. The success of these experiments was confirmed by the identification of pluripotency markers, teratoma formation, chimeras and germline contribution. Additionally, to bypass the innate immune response to viral molecules, a synthetic RNA was successfully developed (Mandal and Rossi 2013). Advances in this methodology may support basic stem biology studies as well as high throughput of the iPSC generation for clinical applications.

Similarly attractive for clinical use are cell-permeable recombinant proteins (Kim et al. 2009), although very low productivity (approximately 0.001 %) remains an obstacle to wider implementation. Zhou et al. (2009), by fusing the C-terminus of four proteins (gene products identified by Yamanaka) with poly-arginine (11R), created recombinant cells which were penetrated with reprogramming proteins. Employing them, the protein-induced PSCs (piPSCs) were generated from murine embryonic fibroblasts. Kim et al. (2009) used the approach to generate stable iPSCs by reprogramming human fibroblasts using OCT4, SOX2, KLF4 and c-MYC proteins fused with cell-penetrating peptides in HEK293 extracts with no further chemical treatment. However, it must be emphasized that this is a challenging method requiring a great deal of laboratory experience and protein chemistry equipment, with a rather low final yield. After comparing different types of iPSC delivery in neuronal differentiation, piPSCs indicated unlimited expansion without cellular senescence (Rhee et al. 2011). However, some remaining undifferentiated cells may induce tumors.

The most recent method with respect to the safety of genetic reprogramming is the small-molecule methodology introduced by Hou et al. (2013), who used seven small-molecule compounds for iPSC generation (yet with low efficiency at level of 0.2 %). This safe method constitutes a promising potential tool for treating diseases and screening systems due to compound permeability, convenient synthesis, preservation, ready access, no immunogenic response, easy handling and reasonable costs (Zhang et al. 2013). The small molecule function may be reversible and was set up by optimizing its optimal concentration. On the other hand, the method is time-consuming for routine use. Reprogramming of mouse cell lasts approximately 40 days; in humans, it is estimated up to 60 days. Despite the lack of mutagenesis, there is still a risk of genetic instability. The epigenetic memory of CiPSCs was also not analysed. Its disadvantage precludes rapid clinical use because of the low efficiency conversion of somatic cells to iPSCs (range 0.1–1 %). To date, successful reprogramming was achieved in mice (Hou et al. 2013). In humans, successful reprogramming without transcription factors was conducted only with cell extracts and/or proteins (Kim et al. 2009; Zheng et al. 2012), but possible clinical advantages may push the technology of human small molecule-derived iPSCs in a relatively short time. Advances in this direction have the potential to provide an unlimited cell supply to regenerative medicine of the desired cell types. It is assumed that potential application will be possible if CiPSCs are generated from patient-specific cells and/or human somatic cells possessing various HLA types at efficiencies exceeding 0.1 % (Higuchi et al. 2014).

To date, a variety of somatic cell types have been selected for successful reprogramming procedures for iPSCs with subsequent differentiation to a broad range of different cells, including germ cells with functional abilities (Aasen and Izpisúa Belmonte 2010; Haase et al. 2009). Skin fibroblasts are the “gold standard” for the technique of genetic reprogramming and any other approaches should be referred to it (Table 5). These cells, as well as keratinocytes, are convenient for non-invasive sampling, which is desired in cellular therapies (Aasen and Izpisúa Belmonte 2010). In this regard, dental pulp stem cells, mesenchymal stromal cells, oral gingival and mucosal fibroblasts, and peripheral blood cells are also advantageous (Egusa et al. 2010; Miyoshi et al. 2010; Oda et al. 2010; Tamaoki et al. 2010). Nonetheless, epithelial cells are supposed to be more susceptible to reprogramming, possibly because of a lack of the mesenchymal-to-epithelial transition. In addition, iPSCs can be derived not only from normal cell types but also from primary cancer and malignant cells. The significant progress made in this field in recent years provides a perspective for a patient-tailored cellular therapy source and a drug testing target without using human embryos and with a limited chance for immune rejection. In addition, the iPSC technique does not require costly and complex equipment and is quite simple to perform.

**Table 5:** Source of cells for induced pluripotent cell generation (based on Li et al. 2014)

Cell type	Skin fibroblasts Keratinocytes	Adipose-derived stem cells	Dermal papilla cells	Blood cells–Hematopoietic stem cells				Urine-derived cells
					Bone marrow	Umbilical cord blood and placenta	Peripheral blood	
Advantages	– Widely used – Well elaborated reprogramming methods	– Readily available – Safe derivation – Lipoaspiration yields variable cell quantities – No necessity for cell expansion	– More effective reprogramming compared to skin or embryonic fibroblasts – High endogenous Sox2 and c-Myc expression	– The most favorable cell source for iPSC induction -–Minimal somatic mutations and –Immunological immaturity in young HSCs	– Abundant HSCs pool	– Abundant HSCs pool – Early developmental origin of CB and placenta – Numerous quantity of cells possessing multilineage differentiation potential – Poorly immunogenic – No ethical concerns – Conceivably low numbers of incorporated mutations	– Minimal risk to the donor when drawing peripheral blood cells with venipuncture – Sufficient amounts with no need to expand in in vitro culture – Convenient access to PBCs in blood banks and retrospective molecular analysis possible in case of rare diseases	– Non-invasive harvesting – Easy to expand – Relatively high reprograming efficiency (to 4 %), also including integration-free method –Theoretically low mutation ratio
Pitfalls	– Biopsy required – Not recommended for patients with severe dermal diseases or burned skin	– Invasive procedure – May be harmful	– Low reprogramming efficiency	– Developmental failures	– Side effects of G-CSF mobilization, e.g. bone pain and nausea – Invasive BM harvesting	– Available only by sampling at birth	– Low HSCs yield – Extremely low efficacy of PBCs reprogramming (at most 0.001 %) – T cell-derived iPSCs may have undesirable germ line IgH and TCR alleles – Possible blood infections – Problematic reprograming for patients with blood disease, e.g. leukemias – Mobilized CD34 + from peripheral blood is problematic, time-consuming and expensive	– High personal variation in number of excreted cells and cell proliferation potential – Long-lasting culturing in vitro – Not well recognized in terms of mutations

*BM* bone morrow, *CB* umbilical cord blood, *G-CSF* granulocyte colony stimulating factor, *HSC* hematopoietic stem cell, *iPSCs* induced pluripotent stem cells, *PBCs* peripheral blood cells

Undoubtedly, one of the troublesome obstacles to implement the iPSC technology in clinical applications, such as personalized cell therapy, is line-to-line iPSC variability resulting in different differentiation fates, tumorigenesis, and the risk of global gene expression depending on particular culture conditions (Newman and Cooper 2010). Also, donor cell types may epigenetically influence the differentiation tendency (Bar-Nur et al. 2011). Moreover, many protocols of iPSC generation have been successful in mice but yield different effects in humans. Thus, some differences between iPSC lines still require documentation of the genetic and epigenetic profiles for appropriate iPSC classification and defining their self-renewal ability, differentiation potential and safety (concerning possible dedifferentiation rate). For instance, some iPSC clones differentiating into a neural lineage were prone to form tumors after transplantation to mouse brains. Gene expression and DNA methylation analysis of undifferentiated cells revealed the activation of genes with long terminal repeats of specific *HHLA1* (human endogenous retrovirus-H LTR-associating 1) sequences (Koyanagi-Aoi et al. 2013). Similarly, an undifferentiated iPSC fraction was identified by Yamashita et al. (2013). During in vitro cartilage tissue engineering, some portion of the iPSCs was left in a pro-oncogenic state and a glandular epithelial tumor formed in mice, in spite of normal morphology, plating efficiency, teratoma forming ability and normal karyotype in in vitro culture. Thus, finding universal safety indicators is a challenge for regenerative medicine utilizing iPSCs in patients.

The answer to these obstacles could be novel strategies of iPSC protocol modifications focusing on the implementation of personalized cellular replacement therapy and/or other biomedical applications, such as disease modelling and pharmaceutical studies. For instance, hepatocytes are considered to be challenging targets for regenerative medicine. This year in the field of hepatology, human self-renewing hepatic lineage-oriented stem cells were generated, which proved the significance of optimizing culture conditions. Modifications significantly determined cellular commitment in vitro. These specific iPSCs autonomously and preferentially differentiate into hepatic-like cells without extra growth factors, chemical compounds and gene transfer (Ishikawa et al. 2015). Currently, research is being conducted in other directions. Recently, human fibroblasts were directly reprogrammed by lentiviral transduction of three selected factors. This technique generated stable lines of human induced hepatocytes that possessed the ability to restore liver function in a mouse model (Huang et al. 2014). Ectopic overexpression of hepatic determination factors along with maturation factors resulted in cells with metabolic activities, hepatotoxin sensitivity and expression profile comparable with primary human hepatocytes (Du et al. 2014). Apart from direct cell lineage conversion successfully changing the fate of somatic cells, another promising approach is establishing homogenous populations of progenitor cells from ESCs or iPSCs. In this way, Cheng et al. (2012) produced non-tumorigenic continuously replicating endodermal progenitor lines with numerous lineage derivatives such as hepatocytes and pancreatic β-cells. They might be a powerful source of undifferentiated tissues, and a more efficient and safer starting point for transplantation therapies. In turn, disappointing results of iPS-derived hepatocytes in terms of human serum albumin production, repopulation of cells in the liver and therapeutic safety prompted some researchers to develop shortcut reprogramming of human fibroblasts omitting the pluripotent intermediate stage (Zhu et al. 2014). Following the procedure, hepatocytes originated from endodermal progenitor cells proliferated extensively in vivo and revealed adult human primary hepatocyte functions.

## Concluding Remarks

PSCs are a huge hope in regenerative medicine in terms of cellular therapy for the treatment of a variety of the diseases of civilization. After cloning Dolly the sheep from a mature somatic cell in 1997, the next breakthrough was the isolation of human ESCs from early embryos and their in vitro culture in a Petri dish. Another milestone in this field was the development of an induced PSC procedure without the necessity of embryos, with the emergence of tremendous potential for the exploitation of autologous stem cells. Recently, the FDA approved the first clinical trials using PSCs in curing macular degeneration (Schwartz et al. 2012) and spinal cord injury (Chapman and Scala 2012). In Japan, the first case of iPSC technology application in the clinic was reported in 2014. In this trial, a RPE cell sheet generated from iPSCs was engrafted to a patient suffering from exudative age-relative macular degeneration (Takahashi 2014). There are still some unsolved aspects for the implementation of the cell therapy. The broad application of PSCs with targeted differentiation can be a powerful tool for studying early embryonic developmental pathways, the etiology of disorders and drug toxicology testing (Rolletschek et al. 2004). Progress in techniques of stem cell (and their descendants) isolation, maintenance and differentiation is a real chance to meet the high expectations of regenerative medicine.
